# Profiling Shoulder Strength in Competitive Surfers

**DOI:** 10.3390/sports6020052

**Published:** 2018-05-30

**Authors:** James Furness, Ben Schram, Tim Cottman-Fields, Brendan Solia, Josh Secomb

**Affiliations:** 1 Water Based Research Unit, Bond Institute of Health & Sport, Bond University, Gold Coast, QLD 4226, Australia; jfurness@bond.edu.au; 2 Faculty of Health Sciences and Medicine, Bond University, Gold Coast, QLD 4226, Australia; timothy.cottman-fields@student.bond.edu.au (T.C.-F.); brendan.solia@student.bond.edu.au (B.S.); 3 Centre for Exercise and Sport Science Research, Edith Cowan University, Joondalup, WA 6027, Australia; joshsecomb37@gmail.com

**Keywords:** surfing, rotator cuff, shoulder, strength ratio, profiling, assessment

## Abstract

The shoulder region has the highest incidence of acute injuries in the sport of surfing. Little is known about the strength profile at the shoulder in a surfing cohort. The primary aim of this study was to establish the reliability of a rotator cuff strength testing procedure for surfers with a secondary aim of providing a profile of internal and external rotation strength in a competitive surfing cohort. Shoulder internal rotation and external rotation isometric strength was measured using a hand-held dynamometer in 13 competitive surfers. Intra-class coefficient values ranged from 0.97 to 0.98 for intra-rater reliability and were lower for inter-rater reliability ranging from 0.80 to 0.91. Internal rotation strength was greater than external rotation strength bilaterally (dominant, *p* = 0.007, non-dominant, *p* < 0.001). No differences (*p* < 0.79) were found in internal rotation strength between the dominant and non-dominant arms. External rotation strength was weaker on the non-dominant arm compared with the dominant arm (*p* < 0.02). The non-dominant arm external rotation to internal rotation ratio (0.82 ± 0.15) was lower (*p* = 0.025) than the dominant arm (0.88 ± 0.14). The current procedure is reliable with the same clinician, and results indicate musculature asymmetry specific to the external rotators.

## 1. Introduction

Over the past 14 years, global involvement in the sport of surfing has more than tripled, from an estimated 13 million participants in 2002 [1] to 37 million recorded in 2013 [2]. It is proposed that this growth in participation will only continue now that surfing has been included for its inaugural appearance in the 2020 Olympics.

Due to the nature of the sporting environment and physical demands, injuries are often inherently associated with participation, with one in every 3 recreational surfers sustaining an acute injury each year [3]. An epidemiology study conducted by Furness et al. [3] found the primary acute injury-prone location was the shoulder (16.4%). This high incidence of shoulder injuries could be attributed to the activity requirements of surfing. Several time motion analysis studies have reported paddling comprised up to 42 to 54% of the total time spent surfing with the average paddling time ranging from 16 to 25 s in duration [4,5,6]. This paddling requirement places significant demand on the shoulders, as the surfer uses an alternate arm action to propel the board forwards. It is proposed that this activity requirement would develop increased shoulder strength and more specifically in muscle groups which extend, abduct and internally rotate the shoulder over opposing muscle groups. However, to the authors’ knowledge, there is no evidence investigating strength profiles in a surfing cohort.

While strength profiles have not been investigated in a surfing cohort, there is increasing evidence to support the association between imbalance or weakness at the shoulder and injury risk in upper arm-dominant sports such as handball, baseball and swimming. A prospective study conducted by Edouard et al. [7] investigated shoulder Internal Rotation (IR) and External Rotation (ER) strength using isokinetic dynamometry in a cohort of female handball players. The study identified that a player who presented with muscular imbalance based on established criteria were two and a half times more likely to suffer a shoulder injury than if that imbalance was not present. Furthermore, Clarsen et al. [8] used a hand-held dynamometer and determined that reduced isometric external rotation weakness was a significant predictor of increased average severity scores related to shoulder injury. Strength profiles have also been assessed in the sport of baseball, which, while being a completely different sport and environment, shares the repetitive internal rotation associated with paddling a surfboard. In baseball studies, reductions in external rotation strength and lower ER/IR ratios have been shown to be associated with shoulder injury [9].

The prospective studies discussed above provide evidence that strength ratios play a role in determining athletes at risk of shoulder injury. In addition to this, several studies have conducted shoulder rotator strength profiling to provide normative baseline data and as a means of tracking changes in muscle groups across a season. For example Ramsi et al. [10] conducted isometric rotator strength profiling across a competitive swimming season and revealed increases in internal rotation strength without equal gains in ER from pre-season to post-season. Hurd et al. [11] conducted a cross sectional study of 165 high school baseball pitchers, providing a strength profile for the internal and external rotators of the shoulder. The authors concluded that this information might be used by clinicians and researchers to interpret muscle strength performance in this population.

It needs to be noted that when strength profiling is conducted at the shoulder, it is done in a sport-specific position, meaning it is similar to how the contractile tissue is stressed during the required activity. For example, the study by Hurd et al. [11] conducted on baseball pitchers utilised a position of testing where the individual was in an upright seated position with the shoulder abducted to 90 degrees, in line with the requirements of pitching. In contrast, the study conducted by Ramsi et al. [10] on swimmers used a prone testing position, with the shoulder abducted to 90 degrees.

Despite shoulder strength ratios being investigated in some sports, there are no studies which assesses shoulder internal or external rotation strength in a surfing cohort. At a minimum it would seem appropriate to establish a rotator strength profile at the shoulder for a competitive surfing cohort to aid clinicians in decision making when treating surfers. Therefore, the primary aim of this study was to establish the reliability of a rotator cuff strength testing procedure for surfers with a secondary aim of providing a profile of internal and external rotation strength in a competitive surfing cohort.

## 2. Materials and Methods

### 2.1. Reliability Phase

Reliability testing was conducted in a control group prior to implementing the testing procedure in a competitive surfing cohort. A total of 21 (18 males and 3 females; 25.29 ± 2.67 years, 80.01 ± 12.43 kg and 177.10 ± 9.02 m) subjects were enrolled to establish intra-rater reliability. Similar sample sizes have been used in both clinical and surf specific studies investigating intra-rater reliability [12,13]. A subset of 12 (9 males and 3 females; 26.00 ± 3.81 years, 78.10 ± 12.57 kg, 177.68 ± 9.47 m) subjects were used to establish inter-rater reliability of the testing procedure. The two physiological movements of IR and ER across the shoulder were examined. The testing order was computer randomised for examiner order, test side (right or left), and movement order. To avoid bias, both examiners and participants were blinded to their own results. A single examiner firstly conducted the entire test battery and was then followed by the alternate examiner. A 5-min rest period was employed during the transition from one examiner to the next, as incorporated by Kelln et al. [14]. The testing methodology including the examiners, equipment and procedure is outlined in the following section and was replicated for the reliability portion of the study.

#### 2.1.1. Subjects

A total of 13 competitive surfers (9 males and 4 females, 24.1 ± 6.9 years, 71.0 ± 8.6 kg and 176.8 ± 5.7 m) comprised the competitive surfing cohort. All surfers were either currently competing or had previously competed at an international level (3 previously competing on the World Qualifying Series and 9 currently competing on the World Qualifying Series and 1 currently competing on the World Championship Tour). All subjects were recruited from the Surfing Australia High Performance Centre and were asked to complete a subjective questionnaire detailing anthropometrics, training habits, surfing history, and injury history prior to undertaking the study. For inclusion within the study, subjects were required to be injury free at the time of testing, be currently engaged in surfing as a primary sport and taking part in competitive surfing.

#### 2.1.2. Examiners

Participant testing was conducted by two Doctor of Physiotherapy students under the supervision of a physiotherapist with 10 years clinical experience. Both examiners underwent five hours of training to ensure familiarisation of the testing technique and data collection device prior to reliability testing. Both phases of the study were approved by the University Human Research Ethics Committee (Approval No: RO1610), and verbal and written consent were obtained.

#### 2.1.3. Equipment

For all strength testing a JTech PowerTrack™ II Commander HHD (JTECH Medical, Salt Lake City, UT, USA) was used. The PowerTrack II™ apparatus includes a force transducer head and attached display panel to view real time data. For each repetition, a ‘make test’ was performed, whereby the examiner holds the dynamometer stationary while the subject exerts a maximal isometric force. Data obtained was then documented as an absolute value of force in Newtons (N).

#### 2.1.4. Testing Positions

**Figure 1:**
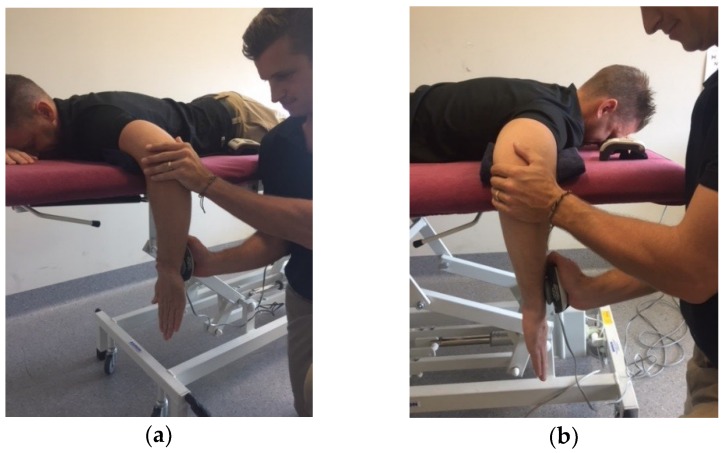
Testing Position: (**a**) Internal rotator strength of the non-dominant shoulder; (**b**) External rotator strength of the dominant shoulder.

The prone testing position used to assess shoulder external and internal rotation isometric strength was adapted from previous research by Ramsi et al. [10] (Figure 1). Subjects were positioned on a height-adjustable plinth in the prone position with the upper arm of tested arm supported by the plinth. The shoulder was positioned in 90° of shoulder abduction and 90° of elbow flexion with an open palm and neutral shoulder rotation. The prone position was employed, as it is representative of the body position utilised throughout the motion of paddling.

The examiner maintained a forward lunge position on the ground with the HHD placed in examiners hand closest to the plinth while testing, with the examiner’s elbow fixed against the anterior aspect of the hip. This position reduces the possibility of the examiner being overcome by the subject and minimizing examiner fatigue. The non-testing hand of the examiner was then used to stabilize the subjects’ elbow to limit compensatory abduction or adduction of the glenohumeral joint. The HHD was placed 2 cm proximal to the ulnar styloid on either the ventral (internal rotation) or dorsal (external rotation) aspect of the subjects’ distal forearm [15].

#### 2.1.5. Testing Procedure

The physiological movement of shoulder IR and ER was assessed in all subjects. The testing order was computer randomised for test side (right or left) and movement order to reduce the influence of fatigue on strength scores.

Measures of moment arm lengths for the shoulder were employed and recorded as a means of further comparative torque (Nm) analysis. Moment arm landmarks and measurements for each movement were measured from the lateral epicondyle to 2 cm proximal to ulna styloid.

To familiarise the subject with the movement, the examiner first passively moved the arm to be tested through the appropriate action and then reassessed the participant complete the movement actively without the HHD to ensure the correct movement was completed. A familiarisation test was then performed, whereby subjects were exposed to identical conditions of a ‘real’ test, however they were only required to perform a submaximal contraction at approximately 50% of Maximal Voluntary Contraction (MVC). This was completed by instructing all participants to contract at half of their maximal effort.

Subjects completed two repetitions for both internal and external rotation. Subjects were instructed to perform the movement and maintain a 3-s sustained maximal isometric contraction against the HHD transducer head. A rest period of 10 s was allowed between each repetition and a 30-s rest between testing of each individual movement (i.e., IR or ER). This protocol was adapted from previous research methods utilizing HHD at the shoulder [15,16].

Verbal instruction and encouragement was standardised across each test for all subjects. The examiner performing the measurement initiated each test with a “1-2-3-go” count. Verbal encouragement of consistent tone and volume with the phrase “push-push-push-push-relax” was provided by the examiner performing the measurement for all subjects.

#### 2.1.6. Data Analysis

Analysis of data was performed using the Statistical Package for the Social Sciences (SPSS Inc. Version 23.0, Chicago, IL, USA). The Intraclass Correlation Coefficient (ICC) was used to reflect the reliability of the measures. Lexell and Downham [17] recommended that ICC values >0.75 represent “excellent reliability” and values between 0.5 and 0.7 indicate “fair to good reliability”. For inter-rater reliability a two-way mixed model was used using average measures of rater 1 and rater 2 (ICC _3,2_). Similarly, intra-rater reliability was determined using a two-way mixed model incorporating single measures obtained by rater 1 (ICC _3,1_). ICC values may be high despite poor trial-to-trial consistency if a high degree of inter-subject variability exists [17]. To negate this issue, the Standard Error of Measurement (SEM) was calculated using the formula $=\sqrt{WMS}$, where *WMS* represents the mean square error from the analysis of variance.

Torque (Nm) was calculated by multiplying the absolute force (N) by average moment arm length for left and right sides (m). Normalised forces (N/kg) and torques (Nm/kg) were determined by dividing the absolute force and torque values by respective participant bodyweights (kg). Shoulder rotation ratios were determined by dividing average external rotation force by average internal rotation force. A single average value for each variable was obtained for the surfing group with males and females combined as a single cohort. Both genders were combined due to the small sample size and were normalised by body weight. Previous research using HHD in overhead athletes has revealed gender differences are absent once normalised to body weight [18].

To test for normality, both a Shapiro-Wilks test (*p* > 0.05) [19] and visual inspection of resulting histograms were conducted within the surfing group. A paired *t*-test was conducted to determine significant differences within the surfing group’s dominant and non-dominant arms, respectively. A Cohen’s *d* effect size was also calculated to reflect the magnitude of any differences identified, with scores greater than 0.8 representing a large effect, 0.5–0.79 representing a moderate effect and 0.2–0.49 a weak effect [20]. Scores between 0.00 and 0.19 represented a trivial effect [21].

## 3. Results

### 3.1. Reliability Phase

Reliability analysis was conducted using ICC and SEM, and the results are presented in Table 1. Relative reliability was expressed using ICC values, which were all within the excellent ranges according to Lexell and Downham [17]. Values ranged from 0.97 to 0.98 for intra-rater reliability and were lower for inter-rater reliability ranging from 0.80 to 0.91. Absolute relative reliability was expressed using SEM which ranged from 7.08 to 7.35 newtons for intra-rater reliability and were higher for inter-rater reliability ranging from 8.88 to 24.00 newtons.

### 3.2. Surfing Cohort

#### 3.2.1. Demographics

Additional information pertaining to surfing experience and training was obtained for the surfing cohort. The average surfing experience, weekly time spent surfing and weekly time spent conducting land-based training was 16.5 ± 7.15 years, 12.0 ± 4.5 h and 4.85 ± 2.66 h, respectively.

#### 3.2.2. Isometric Strength Testing in an Competitive Surfing Cohort

Mean results for both the absolute strength (N) and torque values (Nm) and the normalised force (N/kg) and torque values (Nm/kg) are presented in Table 2. A comparative analysis was conducted between arm dominance for all normalised values using a paired samples *t*-test. When comparing the IR values against the ER values for the same arm, the IR values were significantly higher (dominant, *p* < 0.01, non-dominant, *p* < 0.01). No significant differences were found in IR scores between the dominant and non-dominant arms for normalised force (N/kg) (*p* = 0.79) and normalised torque (Nm/kg) (*p* = 0.81). Significant differences were identified when comparing ER values between the dominant and non-dominant arms with the non-dominant arm being significantly weaker for both normalised force (N/kg) (*p* = 0.02) and normalised torque (Nm/kg) (*p* < 0.01). Further side to side differences were also identified when comparing the ER to IR ratio between the dominant (0.88 ± 0.14) and non-dominant arm (0.82 ± 0.15), with the non-dominant arm revealing a significantly lower ratio when compared to the dominant arm (*p* = 0.03). Table 3 reflects these results for normalised force (N/kg), with the magnitude of the differences expressed as effect sizes. 

## 4. Discussion

The aim of this study was to establish the reliability of a rotator cuff strength testing procedure for surfers and to subsequently provide a profile of internal and external rotation strength in a competitive surfing cohort. The results of this study suggest that this surf-specific measurement procedure displays excellent intra-rater reliability, enabling a rotator cuff strength profile to be developed.

The reliability of the testing procedure was assessed to ensure repeatability prior to implementation of the testing procedure in a surfing population. A prone position was utilised, as this is the primary position in which surfers produce shoulder movement and strength, and surfers can spend up to 54% of a session paddling [5]. The ICC scores produced in the current study for both intra (0.97–0.98) and inter-rater (0.80–0.96) reliability were all above the excellent threshold of 0.75 recommended by Lexell and Downham [17]. The intra-rater ICC scores from the current study are comparable to a study by Holt et al. [16], who also used hand-held dynamometry and assessed IR and ER isometric strength and revealed scores ranging from 0.92–0.96. Regarding inter-rater reliability of the current study, the ICC scores (0.80–0.96) were slightly lower than previous research [15,16], with the 95% confidence intervals indicating wider variability ranging from 0.32–0.99 and larger SEM scores ranging from 9 to 24 newtons. Previous research by Holt et al. [16] revealed higher ICC scores for inter-rater reliability 0.88 to 0.96. One possible rationale for the lower ICC scores in the current study could be associated with the level of experience, as the current study utilised two Doctor of Physiotherapy students and the study by Holt et al. [16] utilised sports physiotherapists with up to 15 years of experience; thus, experience may have assisted with improvements in repeatability. Given the inter-rater results the authors recommend that when using the protocol described within the current study that the same clinician should both assess and monitor an athlete over time.

The secondary aim of this study was to provide a profile for internal and external rotation strength in a competitive surfing cohort. To our knowledge, this is the first study to provide information specific to isometric strength at the shoulder in a surfing cohort and given the activity requirements and high incidence of shoulder injuries, this information may be useful for both enhancing performance and reducing injuries.

The current results revealed normalised strength values of IR and ER isometric strength ranging from 1.6 to 2.1 N/kg within a competitive surfing cohort of both males and females. There are similarities in the current results to previously published normative data studies using HHD. Cools et al. [18] assessed isometric shoulder ER and IR with the shoulder at 90 degrees of abduction and neutral humeral rotation (as per the current study) on 201 upper arm dominant athletes (tennis, volley ball, handball) and determined normalised values ranging from 1.7 to 2.1 N/kg. When closely analysing the data by Cools et al. [18] and inspecting the cohorts with an age range of 18 to 33 years (similar to the current study), the results ranged from 1.8 to 2.1 N/kg for both ER and IR isometric shoulder strength, respectively. While the isometric IR values appear to be identical to the current study, the ER results are consistently lower, regardless of arm dominance (1.8 for non-dominant arms and 1.9 for dominant arms compared with 1.6 in the non-dominant arm to 1.8 in the dominant arm in the surfing cohort).

When further analysing the current study’s ER isometric strength results, there are several similarities to previous research. Firstly, ER results are significantly lower than IR results, regardless of arm dominance. This has been previously reflected in upper arm-dominant athletes such as handball, tennis, volleyball [18], baseball [7] and swimming [10]. When analysing the side to side differences in ER strength, the non-dominant side was significantly weaker (medium magnitude of effect). The previous research reveals mixed results with respect to ER strength and arm dominance, with some studies [11,18] reporting stronger ER in the dominant arm and other studies reporting no difference in ER strength between arms [10,22,23]. It could be argued that this difference is unique to a surfing cohort; however, a larger sample size is needed to confirm this finding.

The IR isometric strength results within the surfing cohort were revealed to be significantly stronger than ER values representing the largest effect sizes (Cohen’s *d* of 1.68 for the non-dominant side and a Cohen’s *d* of 0.90 for the dominant side). This finding of significantly stronger IR values compared with ER values regardless of arm dominance is in agreement with a large portion of research specific to upper arm dominant athletes [10,11,18,23] and in non-athletic populations [23]. This study revealed no differences in IR isometric strength between sides (2.1 N/kg dominant versus 2.1 N/kg non-dominant). This finding is unique to this study, as a large portion of evidence has revealed significant strength differences between sides in upper arm and non-athletic populations [11,18,22,23]. Even in sports such as swimming, where a symmetrical action is performed, differences in IR strength between arms has been identified [10]. The authors propose that this symmetry in IR isometric strength values could be attributed to the activity requirements of paddling, with the surfer utilizing more of a pulling motion and consequently developing musculature which assists with shoulder adduction, extension and internal rotation.

This study was the first to document ER to IR isometric strength ratios at the shoulder in a surfing cohort, with a ratio of 0.88 on the dominant side and 0.82 on the non-dominant side. These ratios appear to be slightly lower than previous research conducted on upper arm-dominant athletes when using a HHD with similar positioning of the shoulder. Previous research has found ER to IR ratios ranging from 0.86 to 1.05 in a cohort of volleyball, tennis and handball athletes [18]. Hurd et al. [11] analysed baseball players and revealed ER to IR ratios ranging from 0.96 to 1.05. Furthermore, the current study revealed the non-dominant arm to have a significantly (*p* < 0.05) lower ER to IR ratio when compared to the dominant arm. This is contrary to previous research conducted in upper arm-dominant athletes, with the dominant arm having consistently higher ER to IR ratios [10,11,18]. The lower IR to ER ratio on the non-dominant side in the current study is due to the weaker external rotators on the non-dominant side, as no side to side differences were detected between internal rotators.

This study has identified symmetry between sides in the internal rotators and significantly weaker external rotators more specifically the non-dominant arm. Given these findings, the authors recommend the described assessment methods to routinely assess athletes who surf. Where asymmetry is identified, specific strengthening exercises should be promoted to address the identified weaknesses. In the case of this study, specific strengthening exercises should be targeted to the external rotators to promote a more symmetrical profile and a ER to IR ratio closer to 1, as asymmetry and lower ER to IR ratios have been identified as risk factors for injury [7,8].

A limitation of this study is the small sample size and the subsequent necessity of combining both females and males into one cohort. To negate the influence of gender differences on strength values data was normalised by weight. Previous research by Cools et al. [18], who also analysed upper arm-dominant athletes and used a similar testing protocol revealed that while males were significantly stronger, when normalised to body weight, differences were absent. Given the small sample size, the findings of this study should not be generalised outside of this current study cohort. Future research is needed within a larger surfing cohort to confirm the current findings and provide more robust recommendations.

## 5. Conclusions

This study has identified a reliable method to assess isometric ER and IR strength when used by the same clinician. The authors recommend this assessment method is used to profile and monitor athletes involved in competitive surfing to assist in their management. Competitive surfers appear to have greater strength in the internal rotator muscle groups compared with the external rotators. Asymmetry was also identified between sides for the external rotators only, with the non-dominant arm being significantly weaker. Coaches and clinicians dealing with surfers should routinely assess isometric strength and where appropriate provide strength training to minimise musculature asymmetry.

## Figures and Tables

**Table 1:** Intra- and Inter-rater reliability for both IR and ER for the non-dominant and dominant arms.

Arm/Movement	Intra-Rater Reliability (*n* = 21)	SEM	Inter-Rater Reliability (*n* = 21)	SEM
Dom IR	0.98 (0.96–0.99)	7.12	0.80 (0.32–0.94)	24.00
Non-Dom IR	0.98 (0.95–0.99)	7.35	0.91 (0.70–0.97)	12.55
Dom ER	0.98 (0.94–0.99)	7.08	0.96 (0.86–0.99)	8.88
Non-Dom ER	0.97 (0.94–0.99)	7.21	0.85 (0.46–0.96)	15.43

*n* refers to the number of subjects, Reliability measures are expressed as Intraclass Correlation Coefficient (95% Confidence Intervals), SEM refers to Standard Error of Measurement, IR refers to Internal Rotation, ER refers to External Rotation, Dom refers to dominant arm, non-dom refers to non-dominant arm.

**Table 2:** Actual and relative mean scores (SD) for both IR and ER for the dominant and non-dominant arms.

Movement	Force (N)	Torque (Nm)	Normalised Force (N/kg)	Normalised Torque (Nm/kg)
IR Dom	148.23 (44.43)	36.56 (12.47)	2.05 (0.43)	0.50 (0.12)
IR Non-Dom	148.50 (34.57)	35.91 (9.64)	2.07 (0.34)	0.50 (0.10)
ER Dom	130.46 (37.84)	32.12 (10.63)	1.81 (0.40)	0.44 (0.11)
ER Non-Dom	118.12 (31.65)	28.55 (8.45)	1.65 (0.34)	0.40 (0.09)

N refers to newtons, Nm refers to newton metres, N/kg refers to newtons per kilogram, Nm/kg refers to newton metres per kilogram.

**Table 3:** Normalised force (N/kg) comparison between dominant and non-dominant arms with associated effect sizes.

Arm/Movement	*p* Value	Effect Size	Magnitude of Effect
IR Dom compared to IR Non-Dom	0.79	−0.07	Trivial
ER Dom compared to ER Non-Dom	0.02	0.76	Medium
IR Dom compared to ER Dom	<0.01	0.90	Large
IR Non-Dom compared to ER Non-Dom	<0.01	1.68	Large
ER/IR Ratio Dom compared to ER/IR Ratio Non-Dom	0.02	0.73	Medium

IR refers to Internal Rotation, ER refers to External Rotation, Dom refers to dominant arm, Non-Dom refers to non-dominant arm.
